# Surveillance of Acute Flaccid Paralysis (AFP) in Greece: 2008–2024

**DOI:** 10.3390/pathogens14100976

**Published:** 2025-09-26

**Authors:** Stavroula Labropoulou, Theano Georgakopoulou, Vahid Baniasadi, Giota Mpizta, Stella Vorre, Maria Theodoridou, Mary Emmanouil, Emmanouil Angelakis

**Affiliations:** 1 National Poliovirus/Enterovirus Reference Laboratory, Hellenic Pasteur Institute, 11521 Athens, Greece; vlabropoulou@pasteur.gr (S.L.); emmanouilm@pasteur.gr (M.E.); 2 Department for Vaccine-Preventable Diseases & Congenital Diseases, National Public Health Organization, 15123 Athens, Greece; t.georgakopoulou@eody.gov.gr (T.G.); s.vorre@eody.gov.gr (S.V.); 3 Diagnostic Department and Public Health Laboratories, Hellenic Pasteur Institute, 11521 Athens, Greece; baniasadi@pasteur.gr (V.B.); giotampizta@pasteur.gr (G.M.); 4 1st Department of Paediatrics, Medical School, National and Kapodistrian University of Athens, 11527 Athens, Greece; mpapagrig@med.uoa.gr

**Keywords:** acute flaccid paralysis, enterovirus, Greece, polio

## Abstract

As part of the WHO’s Global Polio Eradication Initiative, acute flaccid paralysis (AFP) surveillance in children under 15 years old is crucial for monitoring the emergence of polioviruses and tracking Non-Polio Enteroviruses (NPEVs). This study outlines the past 17 years of AFP surveillance in Greece from 2008 to 2024, during which a total of 256 AFP cases were recorded. Stool samples from these cases were analyzed using virus isolation in cell cultures (RD/L20B) and sequencing of NPEV-positive samples. The Attica region reported the highest number of cases with 81 (31%), followed by Central Macedonia and Crete, each with 29 cases (11%). The overall analysis of fecal specimens identified the etiological agent in 18 (7%) specimens, with 13 (4.7%) classified as NPEVs, 4 (1.5%) as adenoviruses, and 1 (0.4%) as a parechovirus. Coxsackievirus A, Coxsackievirus B, and various Echoviruses were the most frequently detected NPEV types. Notably, more than half of these positive specimens (10/18) were from the Attica region, which has the highest population density. These findings highlight the ongoing relevance of AFP surveillance in polio-free settings for broader pathogen monitoring and public health preparedness. Continued vigilance and investment in AFP surveillance are critical to sustaining Greece’s polio-free status and detecting emerging viral threats.

## 1. Introduction

Since the launch of the Global Polio Eradication Initiative (GPEI) at the 41st World Health Assembly in 1988, polio eradication has been a goal of the World Health Organization (WHO) and its member states [1]. The initial aim of eradicating poliovirus-caused poliomyelitis by the year 2000 could not be met; however, since then, there has been more than 99% reduction in annual polio cases worldwide [2]. Greece, along with other WHO European region Member states, has been declared polio-free since 2002, as monitored by the Regional Certification Commission for Polio Eradication (RCCPE) [3].

In addition to routine infant immunization against polio and environmental surveillance of Poliovirus, acute flaccid paralysis (AFP) surveillance in children under 15 years of age is also an essential part of the GPEI strategy for polio eradication [4]. AFP is defined as acute or sudden onset of weakness or paralysis of a limb, characterized by flaccid (reduced tone) in children under 15 years of age [5]. AFP is a clinical syndrome with many infectious and non-infectious causes, including viruses, bacteria, toxins, and systemic diseases. Enteroviruses, flaviviruses and adenoviruses are among the viral pathogens which have been associated with AFP [6].

The annual number of reported AFP cases serves as an indicator of a country’s ability to detect polio, even in countries where the disease no longer occurs. According to WHO recommendations, there are two main AFP quality indicators: the first is ‘non-polio AFP rate’ which indicates that a sensitive surveillance system should detect at least one case of non-polio AFP each year for every 100,000 children under 15 years of age; the second is ‘adequate stool specimen collection’ which requires that ≥80% of AFP cases have two stool specimens collected 24–48 h apart, both within 14 days of paralysis onset, and that these specimens arrive in good condition at a WHO-accredited laboratory [2,7]. AFP surveillance became one of the key polio eradication strategies. It was first established in the Americas during the 1980s and is still being followed today, in close coordination with vaccination programs. Of the 194 WHO member states, 179 conduct AFP surveillance and submit weekly AFP reports to the WHO [7].

Poliomyelitis has been classified as a notifiable disease in Greece since 1950. Mandatory surveillance of AFP was introduced in September 1996. In alignment with the WHO guidelines issued in 1997, the National Public Health Organization (NPHO) established a clinical and laboratory-based AFP surveillance network in March 1998 and implemented a comprehensive set of poliomyelitis surveillance measures across Greece: The NPHO conducts intensive, weekly active surveillance for AFP at the national level. Healthcare professionals—including neurologists, pediatricians, and ICU physicians—report AFP cases to NPHO and submit stool samples to the National Poliovirus/Enterovirus Reference Laboratory at the Hellenic Pasteur Institute. Since 2012, targeted stool sample collection has also been carried out among high-risk groups such as the Roma community and refugees/migrants. In addition, sewage samples are collected from wastewater treatment plants in areas with high-risk populations and tested for wild poliovirus. As of 2024, urban wastewater has also been incorporated into the environmental surveillance strategy. Since 2008, the National Poliovirus/Enterovirus Reference Laboratory of the Hellenic Pasteur Institute has conducted supplementary surveillance of circulating enteroviruses in fecal specimens and environmental samples [4,8,9]. Greece has reported no cases of wild poliovirus since 1997, a testament to the country’s high vaccination coverage and strong population immunity. Between 1998 and 2002, four cases of vaccine-associated paralytic poliomyelitis (VAPP) were documented, linked to the use of the oral polio vaccine (OPV). From 2002 to 2025, no cases of wild poliovirus or circulating vaccine-derived poliovirus (cVDPV) have been reported to the NPHO’s Department for Vaccine-Preventable Diseases. These findings affirm Greece’s continued status as a polio-free country [10]. This study investigates the AFP surveillance status in Greece over the past 17 years.

## 2. Materials and Methods

### 2.1. Specimens

From January 2008 to December 2024, stool specimens from 256 children diagnosed with AFP were received by the National Poliovirus/Enterovirus Reference Laboratory for the diagnosis of a possible viral etiology of the disease. A second sample was collected within 48 h of the first sample, and a third sample was collected 60 days later. The specimens were sent from hospitals across different regions of Greece and arrived in good condition, in accordance with WHO guidelines (Good condition: reverse cold chain maintained and received without leakage or desiccation).

### 2.2. Treatment of Clinical Samples

Collected stool specimens were stored at −20 °C, prior to treatment. Two grams of each sample were homogenized in a mixture consisting of sterile glass beads (VWR International GmbH, Wien, Austria) 90% Dulbecco’s modified Eagle medium (DMEM) (GIBCO, Grand Island, New York, NY, USA), and 10% chloroform (AppliChem, GmbH, Darmstadt, Germany). The homogenized mixture was then centrifuged at 2000 rpm for 20 min. Viral RNA was isolated from 140 μL of the supernatant using QIAamp viral RNA minikit (Qiagen, Hilden, Germany), following the manufacturer’s instructions.

### 2.3. Molecular Detection of Enterovirus

The primers and probes used in these experiments for enterovirus detection were employed as previously described [11]. The Poliovirus rRT-PCR ITD 5.2 Kit (Centers for Disease Control, Atlanta, GA, USA) was used to specifically detect PVs in stool specimens, and intratypic differentiation between WPV and SL strains was performed using the Poliovirus rRT-PCR VDPV 6.0 Kit (Centers for Disease Control, Atlanta, GA, USA). To prevent template loss and contamination, the potato Solanum tuberosum phyB gene was co-purified and co-amplified with each sample, as previously described [12]. Strict laboratory practices, including triple room separation, were implemented to avoid PCR contamination.

### 2.4. Cell Cultures, Viral Isolation, and Serotyping

Stool specimens were inoculated into the rhabdomyosarcoma (RD) cell line, a human cell line that supports the replication of most EV strains. Additionally, a genetically engineered mouse cell line (L20B), which stably expresses the human poliovirus receptor, was also used for the inoculations. The cell lines were kindly provided by the WHO regional reference laboratory (Rome, Italy) and were used in accordance with WHO guidelines [13]. Cell cultures were monitored for visible cytopathic effects (CPE). In RD cells in which cytopathic effect (CPE) was observed, the cells were harvested and kept at −20 °C for further analysis.

Enterovirus serotyping was performed on isolates derived from EV-cell culture by using pooled antisera produced against PV and CVB in seroneutralization assays. Echovirus serotyping was carried out utilizing A-G antisera pools (RIVM, the Dutch National Institute for Public Health and the Environment) in accordance with WHO guidelines [13].

### 2.5. Enterovirus Genotyping

The VP1 region was amplified using semi-nested RT-PCR for genotypic identification of enterovirus strains. In brief, RNA isolation was followed by cDNA synthesis and a semi-nested RT-PCR, as described previously [14]. The PCR products were purified using the QIAquick PCR purification kit (Qiagen, Hilden, Germany) and the MinElute gel ex-traction kit (Qiagen). The purified products were sequenced bidirectionally by performing Sanger sequencing with the BigDye Terminator v3.1 Cycle Sequencing Kit on an Applied Biosystems 3500 Genetic Analyzer (Applied Biosystems, Waltham, MA, USA). The electrochromatography data of sequencing were analyzed with Nucleotide BLAST online version “https://blast.ncbi.nlm.nih.gov/ (accessed on 15 July 2025)” and predicted genotypes were confirmed using the Enterovirus Genotyping Tool v0.1 “https://mpf.rivm.nl/mpf/typingtool/enterovirus/ (accessed on 15 July 2025)” [15,16].

## 3. Results

### 3.1. Patients, Clinical History and Vaccination Coverage

**Figure 1:**
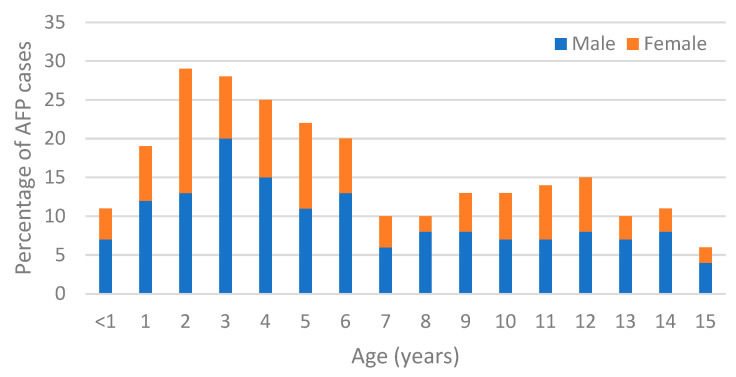
Percentage of AFP incidence and age distribution.

A total of 256 stool specimens from children with AFP symptoms were analyzed. Out of these, 154 (60%) were from male and 102 (40%) were from female patients. Age distribution revealed that 123 (48%) children were between 1 and 5 years of age and 11 (4%) were less than one year old (Figure 1 and Table 1). Overall, a high level of vaccination coverage was observed throughout the years. A total of 211 (82%) children had a history of vaccination with inactivated polio vaccine (IPV), 13 (5%) had received oral polio vaccine (OPV), and 9 (3.5%) had received a combination of OPV and IPV. The clinical history of the patients indicated that 41 (16%) experienced fever at the onset of paralysis, 45 (18%) exhibited asymmetric paralysis, and 183 (71%) had paralysis progression within three days (Table 1). Follow-up assessments indicated that 65% of patients recovered fully with no residual weakness.

### 3.2. AFP Surveillance Indicators

The expected number of AFP cases was determined based on the WHO guideline that mandates reporting at least one case of AFP per 100,000 children under the age of 15. Population statistics for Greece were obtained from the Hellenic Statistical Authority “www.statistics.gr (accessed on 21 March 2025)” and showed a gradual decline in the number of children within this age group over the study period. Data is presented in detail in Table 2. The AFP rate was calculated annually by dividing the number of reported cases by the expected number. Overall, the AFP rate met the WHO target in approximately half of the study years, with notable disruptions during the COVID-19 pandemic. According to WHO criteria, adequate stool specimens’ collection requires that at least 80% of AFP cases have two stool specimens collected 24–48 h apart, both within 14 days of paralysis onset. Additionally, a third sample should be collected 60 days after onset for follow-up. In most years both stool specimen adequacy and 60-day follow-up exceeded WHO targets. Although surveillance indicators mostly met WHO targets, the onset of the COVID-19 pandemic in 2019 posed significant challenges in meeting these standards during the following two years (Table 2).

### 3.3. EV Isolation and Typing

**Figure 2:**
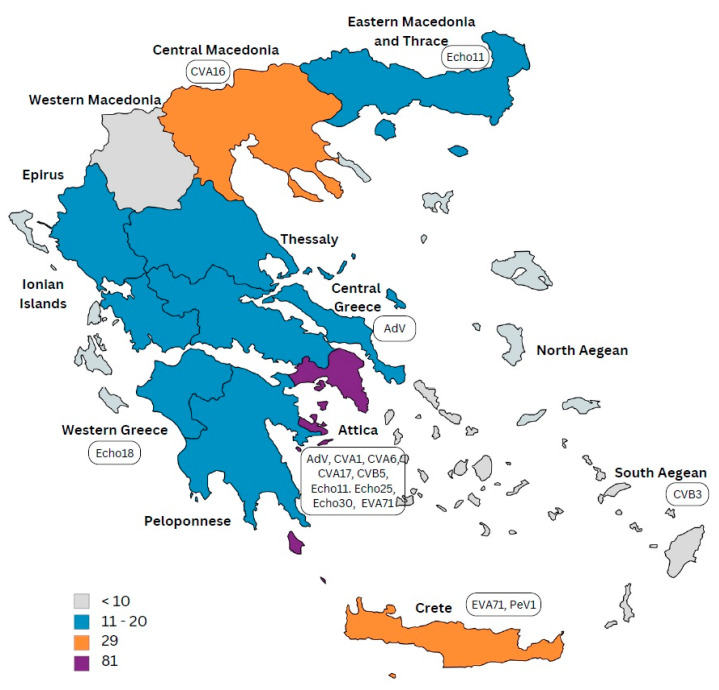
Geographical distribution of the number of AFP cases and virus genotypes in Greece. The map was created with the MapChart online version “www.mapchart.net (accessed on 10 April 2015)”.

Stool specimens (first and second samples) were analyzed from 256 AFP patients and in all cases that both samples were adequate, detection of viral pathogen was confirmed in both samples. We identified 13 NPEV, four adenovirus infections (from 2011 until 2015), and one parechovirus case in 2015 (Table 3). The 60-day follow up samples were collected in 89% of the cases (Table 2) and were all found negative for enteroviral infection. During the 17-year study period, no enterovirus was detected in seven of those years. The majority of detected NPEVs belonged to Enterovirus B group (CoxB3, CoxB5, Echo 11, Echo 18, Echo 25, and Echo 30), which were detected from 2008 to 2023. In contrast, Enterovirus A strains (CoxA6, CoxA16 and EVA71) were found between 2013 and 2019, while Enterovirus C strains (CoxA1 and CoxA17) were found only in the years 2013 and 2014. Notably, genotyping and serotyping were conducted for positive samples collected before 2017, while only genotyping was performed for positive samples collected after 2017. More than half (10/18) of the positive stool specimens were from the Attica region, which has the highest population density (Figure 2).

### 3.4. AFP Distribution

The Attica region, which includes the capital Athens and has the highest population density, reported the largest number of AFP cases, with 81 cases (31%) over the 17-year period. Central Macedonia and Crete followed, each accounting for 29 cases (11%) (Figure 2). Annual case counts fluctuated over time, with most regions showing sporadic incidence. Notably, some regions (e.g., Western Macedonia, Ionian Islands) had multiple years with zero reported cases, while Attica reported cases in nearly every year of surveillance. Overall, the lowest number of cases were reported from the islands and the Western Macedonia region, while other regions of Greece reported 10–20 cases annually. Detailed annual incidence per region is shown in Supplementary Table S1.

## 4. Discussion

Despite the WHO polio eradication campaign, which is trying to achieve its goal through global immunization efforts, sporadic reports of polio infections still emerge in Afghanistan and Pakistan. Therefore, AFP surveillance remains a critical complement to polio vaccination strategies [4,17]. Although Europe has been declared polio free for more than two decades, active AFP surveillance among children is essential for ensuring the vigilance of the countries’ surveillance system [3]. Given the numerous reports on AFP surveillance in Europe and other parts of the world [4,6,17,18,19,20,21,22,23,24,25], it is equally important to understand the status of AFP surveillance in Greece, which is a country located on major immigration routes [26].

In this study, we present a comprehensive overview of AFP surveillance in Greece over the past 17 years (2008–2024) and assess its quality based on WHO surveillance performance indicators [2]. The age distribution of children with AFP in Greece showed that the majority of AFP cases occurred in children aged 1–5 years (48%), a pattern similar to findings from other countries and reflects the age group most vulnerable to enteroviral infections and poliovirus-like syndromes [4,18,21,25]. Vaccination coverage remained generally high, with 88% of children having received at least three doses of IPV or OPV. However, approximately 9% had unknown or incomplete vaccination histories, emphasizing the need to enhance record-keeping and target under-immunized populations, particularly among marginalized or migrant communities [26]. While surveillance indicators mostly met WHO targets, the onset of the COVID-19 pandemic in 2019 made it difficult to meet these standards for the next two years. Nevertheless, follow-up at 60 days post-onset of paralysis remained strong in most years, suggesting ongoing commitment to case tracking even under strained conditions. This resilience highlights the critical importance of maintaining robust infrastructure and human resources dedicated to public health surveillance systems, even during crises. The geographical distribution of AFP cases correlates with the population density of Greece. The Attica region, which hosts more than 40% of the population, reported 81 (31%) AFP cases, followed by Central Macedonia and Crete with 29 cases (11%) each. These findings emphasize the importance of surveillance by geographic and demographic parameters to ensure representative coverage across the country. Interestingly, more than half of the virus-positive AFP cases originated in the Attica region. This might reflect a combination of higher transmission potential due to dense population, better healthcare access and diagnostic facilities, or more rigorous reporting practices.

Although poliovirus was not isolated in any of these cases—supporting Greece’s polio-free status—non-polio enteroviruses (NPEVs) and other viral agents such as adenoviruses and parechoviruses were detected, affirming the continued relevance of AFP surveillance not only for poliovirus detection but also for broader viral pathogen monitoring. While only 7% (18/256) of AFP cases yielded an identifiable viral cause, these results are consistent with other studies where viral identification rates range from 5 to 15%, depending on laboratory capacity, sample timing, and methodology [23,27]. The majority of identified enteroviruses belonged to Enterovirus B species, consistent with previous Greek and international studies [9,25,28]. These viruses, including Coxsackie B and echoviruses, are well-known causes of viral meningitis and myelitis and can mimic poliomyelitis. Enteroviruses A and C were mainly detected between 2013 and 2019, which may reflect temporal shifts in circulating serotypes, possibly influenced by population immunity, seasonal factors, or regional outbreaks. Adenoviruses and parechoviruses, although less commonly linked to AFP, were also identified, reaffirming that the clinical syndrome has a broad etiological spectrum and necessitates comprehensive laboratory testing [5,6]. Interestingly, in one of the children with AFP symptoms (a two-year-old boy), in which no viral agent could be detected in his stool specimens, further analysis revealed the presence of West Nile virus IgG antibodies in serum along with IgM antibodies in both serum and cerebrospinal fluid [29].

## 5. Conclusions

Seventeen years of acute flaccid paralysis (AFP) surveillance in Greece have demonstrated the country’s ongoing commitment to maintaining a high-quality system aligned with WHO performance indicators. Even amid challenges such as the COVID-19 pandemic, Greece sustained essential surveillance standards, especially in stool specimen adequacy and 60-day follow-ups. The consistent detection of non-polio enteroviruses (NPEVs), adenoviruses, and other pathogens underscores the relevance of AFP surveillance not only for poliovirus monitoring but also for broader viral pathogen detection. The findings reinforce the value of integrating clinical, epidemiological, and laboratory data to ensure rapid identification and response to emerging threats. Continued investment in surveillance infrastructure, vaccination coverage, and targeted efforts in under-immunized or migrant populations remain critical to safeguarding Greece’s polio-free status and strengthening its public health defenses.

## Figures and Tables

**Table 1:** Demographic and clinical characteristics of AFP cases reported in Greece.

	2008	2009	2010	2011	2012	2013	2014	2015	2016	2017	2018	2019	2020	2021	2022	2023	2024	Total
Number of AFP cases	18	16	20	24	16	23	15	18	18	10	17	11	7	4	18	9	12	256
Gender																		
Male	10 (56%)	14 (88%)	13 (65%)	17 (71%)	10 (63%)	13 (57%)	10 (67%)	6 (33%)	10 (56%)	5 (50%)	11 (65%)	6 (55%)	6 (85%)	1 (25%)	9 (50%)	6 (67%)	7 (60%)	154 (60%)
Female	8 (44%)	2 (12%)	7 (35%)	7 (29%)	6 (37%)	10 (43%)	5 (33%)	12 (67%)	8 (44%)	5 (50%)	6 (35%)	5 (45%)	1 (15%)	3 (75%)	9 (50%)	3 (33%)	5 (40%)	102 (40%)
Age (years)																		
<1	2 (11%)	2 (12%)	0 (0%)	2 (8%)	0 (0%)	0 (0%)	0 (0%)	0 (0%)	2 (11%)	0 (0%)	0 (0%)	1 (9%)	0 (0%)	0 (0%)	1 (6%)	0 (0%)	1 (8%)	11 (4%)
1–5	12 (66%)	4 (25%)	11 (55%)	15 (63%)	7 (44%)	13 (57%)	8 (53%)	9 (50%)	7 (39%)	4 (40%)	7 (42%)	5 (46%)	3 (42%)	2 (50%)	6 (33%)	4 (44%)	6 (50%)	123 (48%)
6–10	3 (17%)	7 (44%)	3 (15%)	4 (17%)	4 (25%)	7 (30%)	4 (27%)	5 (28%)	7 (39%)	4 (40%)	5 (29%)	4 (36%)	2 (29%)	1 (25%)	2 (11%)	1 (12%)	3 (25%)	66 (26%)
11–15	1 (6%)	3 (19%)	6 (30%)	3 (12%)	5 (31%)	3 (13%)	3 (20%)	4 (22%)	2 (11%)	2 (20%)	5 (29%)	1 (9%)	2 (29%)	1 (25%)	9 (50%)	4 (44%)	2 (17%)	56 (22%)
Vaccination history																		
0 or unknown dose	2 (11%)	7 (44%)	3 (15%)	2 (8%)	0 (0%)	0 (0%)	0 (0%)	0 (0%)	0 (0%)	0 (0%)	1 (6%)	0 (0%)	0 (0%)	0 (0%)	6 (33%)	1 (11%)	1 (8%)	23 (9%)
1–2 doses	1 (6%)	0 (0%)	0 (0%)	0 (0%)	0 (0%)	2 (9%)	1 (7%)	0 (0%)	1 (6%)	1 (10%)	0 (%)	1 (9%)	0 (0%)	0 (0%)	0 (0%)	1 (11%)	1 (8%)	9 (3%)
3 or more doses	15 (83%)	9 (56%)	17 (85%)	22 (92%)	16 (100%)	21 (91%)	14 (93%)	18 (100%)	17 (94%)	9 (90%)	16 (94%)	10 (91%)	7 (100%)	4 (100%)	12 (67%)	7 (78%)	10 (84%)	224 (88%)
Clinical history																		
Fever at onset	4 (22%)	3 (19%)	3 (15%)	3 (13%)	0 (0%)	2 (9%)	3 (20%)	2 (11%)	7 (39%)	0 (0%)	4 (24%)	2 (18%)	0 (0%)	0 (0%)	6 (33%)	2 (22%)	0 (0%)	41 (16%)
Paralysis progression	17 (94%)	12 (75%)	13 (65%)	21 (88%)	14 (88%)	18 (78%)	11 (73%)	13 (72%)	9 (50%)	6 (60%)	13 (76%)	6 (55%)	6 (86%)	1 (25%)	11 (61%)	5 (55%)	7 (58%)	183 (71%)
Asymmetric paralysis	3 (17%)	2 (12%)	6 (30%)	4 (17%)	3 (19%)	4 (17%)	1 (7%)	0 (0%)	3 (17%)	0 (0%)	3 (18%)	0 (0%)	1 (14%)	2 (50%)	9 (50%)	3 (33%)	1 (8%)	45 (18%)

**Table 2:** AFP surveillance performance indicators in Greece (Bold = meeting WHO target). Non polio AFP rate per 100,000 children under the age of 15; two stool specimens collected 24–48 h apart within 14 days of onset of paralysis and adequately shipped to the laboratory; Surveillance Index = non-polio AFP rate (not to exceed the target) × percent adequate stool collection; AFP cases follow up after 60 days.

	2008	2009	2010	2011	2012	2013	2014	2015	2016	2017	2018	2019	2020	2021	2022	2023	2024
Number of expected cases in <15 children	17	17	17	17	17	17	16	16	16	16	16	16	16	15	15	14	14
Number of AFP cases	18	16	20	24	16	23	15	18	18	10	17	11	7	4	18	9	12
Non polio AFP rate	**1.05**	0.94	**1.17**	**1.41**	0.94	**1.35**	0.94	**1.12**	**1.1**	0.63	**1.06**	0.69	0.44	0.27	**1.2**	0.64	0.86
Adequate specimen	**94%**	**88%**	65%	**88%**	**100%**	**91%**	**100%**	**94%**	**100%**	**100%**	**94%**	**100%**	**85%**	**100%**	72%	56%	75%
Surveillance index	**0.94**	**0.82**	0.65	**0.88**	**0.94**	**0.91**	**0.94**	**0.94**	**1**	0.63	**0.94**	0.69	0.38	0.27	0.72	0.35	0.65
60-day follow-up	**89%**	**100%**	**100%**	**96%**	**100%**	**96%**	**87%**	**100%**	**100%**	**100%**	**100%**	**100%**	**85%**	25%	**100%**	78%	58%

**Table 3:** Viral agent detection in the stool specimens of children diagnosed with AFP.

	2008	2009	2010	2011	2012	2013	2014	2015	2016	2017	2018	2019	2020	2021	2022	2023	2024
Nο. of AFP cases	18	16	20	24	16	23	15	18	18	10	17	11	7	4	18	9	12
Enterovirus cause	1	0	1	1	0	2	2	1	2	0	0	1	0	0	1	1	0
NPEV type	Echo 30	-	CoxB3	Echo 11	-	CoxA17 CoxA6	Echo 25 CoxA1	Echo 18	EVA71	-	-	CVA16	-	-	CoxB5	Echo 11	-
Other causative	-	-	-	3	-	-	1	2	-	-	-	-	-	-	-	-	-
Type				2 Adenoviruses, West Nile virus			Adenovirus	Adenovirus Parechovirus									

## Data Availability

The original contributions presented in the study are included in the article/Supplementary Materials, and further inquiries can be directed to the corresponding author.

## Supplementary Materials

The following supporting information can be downloaded at: https://www.mdpi.com/article/10.3390/pathogens14100976/s1, Table S1: AFP cases in different districts of Greece.
