# Supplementary material for: Surveillance of Acute Flaccid Paralysis (AFP) in Greece: 2008–2024
Source: Pathogens. 2025 Sep 26;14(10):976. doi: 10.3390/pathogens14100976 (PMC12567247; doi:10.3390/pathogens14100976)
Supplement: Supplementary file 1 [file pathogens-14-00976-s001.zip › pathogens-3884771-supplementary.pdf]

	2008	2009	2010	2011	2012	2013	2014	2015	2016	2017	2018	2019	2020	2021	2022	2023	2024	Total
Eastern Macedonia & Thrace	1	-	1	1	1	2	1	3	1	2	-	1	1	1	2	2	-	20
Attica	6	4	6	10	7	7	5	4	7	1	9	-	2	-	6	2	4	81
Western Greece	1	3	3	1	-	3	1	1	-	1	-	1	-	1		-	-	16
Western Macedonia	-	-	-	-	-	-	-	-	-	1	-	-	-	-	2	-	1	4
Ionian Islands	1	1	-	-	-	-	-	-	-	-	-	-	1	-	-	-	-	3
Epirus	1	1	-	1	1	1	2	1	1	-	1	-	-	1	-	-	1	12
Central Macedonia	2	-	1	3	4	3	1	3	4	-	1	3	2	-	-	1	2	29
Crete	3	2	3	3	1	1	1	3	4	3	-	1	-	-	2	-	2	29
South Aegean	-	1	2	1	1	1	1	-	-	1	1	-	-	-		-	-	9
Peloponnese	2	2	1	2	-	1	-	-	-	1	-	-	-	-	2	1	-	12
Central Greece	-	1	-	-	-	2	2	1	1	-	2	3	1	-	1	-	1	15
Thessaly	-	-	3	2	1	1	-	2	-	-	2	1	-	-	3	3	1	19
North Aegean	1	1	-	-	-	1	1	-	-	-	1	1	-	1	-	-	-	7

Table S1. AFP cases in different districts of Greece.
